# Gone Too Soon: Higher Pretreatment and Treatment Dropout Among Emerging Adults in a Women‐Specific Outpatient Treatment Service for Substance Use Disorders in Brazil

**DOI:** 10.1111/eip.70133

**Published:** 2026-01-11

**Authors:** Isadora Espíndola Leite Borges, Gabriel dos Reis Stafuzza, Pedro Luis Freitas Tótolo, Mario Nicolau Silva Gomes, Eduardo Janotti Cavalcante, Paulo Jeng Chian Suen, Andreza Aparecida Miranda dos Santos, Pedro Mario Pan, Silvia Brasiliano, Patrícia Brunfentrinker Hochgraf, Pedro Starzynski Bacchi

**Affiliations:** ^1^ School of Medicine, University of São Paulo São Paulo Brazil; ^2^ Institute of Mathematics and Statistics of the University of São Paulo São Paulo Brazil; ^3^ Institute of Psychiatry, Hospital das Clínicas, University of São Paulo Medical School São Paulo Brazil; ^4^ Laboratory of Integrative Neuroscience (Linc), Department of Psychiatry Federal University of São Paulo São Paulo Brazil; ^5^ Laboratory of Psychiatric Neuroimaging (LIM‐21), Department and Institute of Psychiatry Hospital das Clínicas, University of São Paulo Medical School São Paulo Brazil

**Keywords:** alcohol‐related disorders, Brazil, gender equity, substance‐related disorders, women, young adult

## Abstract

**Aim:**

Emerging adulthood is a critical period for health interventions. The gender gap in substance use disorder (SUD) prevalence between men and women has been consistently narrowing, particularly amongst emerging adults (EAs), yet women remain underrepresented in SUD research. This study aims to investigate pretreatment and treatment dropout rates and predictors in patients at a Women‐Specific Outpatient Service in Brazil, across different age strata: emerging adults (EAs, 18–25), middle aged adults (MAAs, 26–59), and older adults (OAs, 60+).

**Methods:**

An observational cohort study across 27 years. Pretreatment dropout was evaluated with multivariate logistic regression. Survival curves with dropout as event were estimated for each age stratum and log rank tests were performed. Survival probabilities and Cox proportional hazards models were used to gauge dropout risk and determine dropout risk factors.

**Results:**

The 756 women were included, comprising 125 EAs, 585 MAAs, and 46 OAs. MAAs showed lower odds of pretreatment dropout than EAs (OR = 0.56, *p* = 0.025). EAs exhibited lowest treatment retention, with only 13% remaining in treatment at 104 weeks (*p* < 0.0001). Cox regression indicated that higher education was protective, with tertiary education reducing dropout risk (HR = 0.65, *p* = 0.038), whilst cocaine/crack use increased it (HR = 1.57, *p* = 0.017). Patients enrolled in more recent years had a higher risk of dropout whithin the first six months of treatment.

**Conclusions:**

Emerging adulthood is a critical period for health interventions. The gender gap is narrowing amongst EAs, and women are still underrepresented in studies. We found very high pretreatment and treatment dropout rates amongst Emerging Adults in our sample. These findings underscore the necessity for early‐stage risk stratification and intervention protocols for this subpopulation.

## 1 Introduction

Substance use disorders (SUDs) represent a major public health issue, significantly impacting both mental and physical health and contributing to substantial social costs globally (Griswold et al. 2018). Alcohol use prevalence stands at more than 43% worldwide (World Health Organization 2018), whilst 296 million people aged 15–64 reported using illicit or non‐prescribed drugs in 2022 (United Nations Office on Drugs and Crime 2022). Although SUDs have historically been considered more prevalent amongst men, the gender gap is narrowing. In the United States, the male‐to‐female ratio for alcohol use disorders (AUDs) was around 5:1 in the 1980s, but recent studies indicate it has shifted to approximately 1.5:1 (Grant et al. 2017; McHugh et al. 2018). In Brazil, data from the 2019 National Health Survey (PNS) indicated that 37.3% of men and 17.1% of women consumed alcoholic beverages at least once a week. Between 2013 and 2019, there was a significant increase in heavy drinking behaviour, particularly amongst younger individuals, white and brown populations, single individuals, and women aged 30–44 years (Plens et al. 2022). The Brazilian healthcare system for substance use disorders is primarily provided through Psychosocial Care Units (CAPS‐AD), a free service covered by Brazil's unified public health system, offering multidisciplinary professional care primarily focused on psychosocial interventions. The patients are predominantly men, with alcohol and cocaine/crack being the most commonly used substances. The main objective of CAPS‐AD has been to implement community‐based care services for mental health and substance use, facilitating the deinstitutionalization of psychiatric hospitals (Gallassi et al. 2016).

Emerging adulthood has been conceptualised as a specific developmental period between the ages of 18 and 25, with distinct biopsychosocial characteristics that may influence substance use, treatment‐seeking, and retention (Arnett 2000; Dayal and Balhara 2017). In the United States, evidence indicates that the gender gap in Alcohol Use Disorder is narrowing, particularly amongst emerging adults (EAs) and adults. Some studies suggest that this trend is likely due to an increase in women starting alcohol use whilst rates amongst men have stabilised (Keyes et al. 2008; White 2020). Most individuals undergoing treatment for drug use disorders in Africa and Latin America are under 35 years old (Goodman et al. 2015). Results from the 2010 National Survey on Drug Use and Health (NSDUH) point out that, within the specific age group of 18 to 25, the rate of past month illicit drug use was 21.5%, compared to only 6.6% amongst adults aged 25 and older (McCance‐Katz 2019). Evidence suggests that EAs have lower retention rates in treatment, with contributing factors including reduced motivation for abstinence, lower readiness to change, higher psychiatric comorbidity, increased social pressures, demographic instability, identity exploration, and a greater susceptibility to engaging in risky behaviours (DiClemente et al. 2009; Satre et al. 2003, 2004; Schuman‐Olivier et al. 2014; Smith et al. 2010, 2011; Welsh et al. 2019).

Substance use treatment completion is strongly associated with favourable outcomes, such as long‐term abstinence and/or substance use reduction, improved employment rates, and reduced criminal behaviour (Dalton et al. 2021; Momen et al. 2020; Stark 1992; Timko et al. 2016). A systematic review of 124 studies, involving 199 617 participants, found that dropout rates are highly variable across different treatment settings and methodologies, with some programmes experiencing dropout rates as high as 67.7% (Brorson et al. 2013). A small number of individual‐level factors, such as younger age, lower cognitive functioning, and personality disorders, have been consistently identified as risk factors for dropout (Brorson et al. 2013), and the age group of EAs is particularly susceptible (Adams et al. 2017; Arnett 2000; Smith et al. 2014).

Findings regarding gender differences in SUD treatment retention are conflicting (Brasiliano et al. 2020; Greenfield et al. 2007; Grosso et al. 2013; Verissimo and Grella 2017). Given the alarming changes in substance use patterns amongst EAs and the underrepresentation of women in both research and mixed‐gender treatment programmes (Canada. Health Canada 2001; Greenfield et al. 2007; Lal et al. 2015), there is an urgent need to understand the challenges in treatment retention amongst female EAs. This group represents a population with significant potential for early intervention. Therefore, we further examined treatment retention in the Women Drug Dependent Treatment Center (PROMUD), a gender‐specific outpatient treatment center in Brazil. Our aims were threefold:

1. To understand predictors of pretreatment dropout, our hypothesis was that emerging adults would have a lower likelihood of attending their first appointment of treatment.
2. To compare treatment retention across age strata: emerging adults (EAs; 18–25 years), middle aged adults (MAAs 26–59 years), and older adults (OAs; 60+ years). We hypothesized that the emerging adults would have lower treatment retention rates compared to older populations, as previously reported (Dalton et al. 2021; Dayal and Balhara 2017; Schuman‐Olivier et al. 2014).
3. To assess whether sociodemographic and clinical variables—such as race, education level, income, marital status, previous suicide attempts, sexual orientation, history of sexual violence, having children, year at admission, and primary substance of choice—would be associated with dropout risk in the treatment group.

## 2 Materials and Methods

### 2.1 Study Setting and Design

This observational cohort study was conducted at PROMUD (Women Drug‐Dependent Treatment Center), based at the Institute of Psychiatry of the University of São Paulo Medical School Clinical Hospital (IPq‐HC/FMUSP). PROMUD is a pioneering outpatient treatment center dedicated exclusively to women with SUD, providing high‐level tertiary and quaternary mental health care. Established in 1995, PROMUD was specifically designed to address the unique sociodemographic and substance use profiles of women (Hochgraf and Andrade 1995). Further details about the center's structure and operations can be found in previous publications (Brasiliano et al. 2020; Brasiliano and Hochgraf 1999; United Nations Office on Drugs and Crime 2004; Figueiredo et al. 2024).

The treatment at PROMUD follows a standardised protocol (Brasiliano and Hochgraf 1999) and is based on a primarily outpatient model, structured around a continuing care approach. A treatment period of two years was established to support sustained abstinence and provide comprehensive, multicomponent interventions, including pharmacological treatment, psychological support, nutritional and occupational therapies, and legal counselling. The care team comprises psychiatrists, psychologists, family therapists, nutritionists, and legal professionals, offering individualised interventions tailored to patients' comorbidities and psychosocial needs. Core components include psychoanalytically oriented group therapy, psychiatric monitoring with evidence‐based pharmacological management, family therapy, and legal assistance related to family law. After one to two years of treatment, patients are discharged in improved health and transitioned to primary care services for continued management of chronic conditions and participation in relapse prevention programmes. Hospitalisation is reserved only for patients with severe clinical and/or psychiatric complications or when outpatient treatment proves ineffective. Patients are admitted if they meet the service's inclusion and exclusion criteria, through an interview in intake. If a patient misses an appointment, she is contacted by phone or written letter for three consecutive weeks. Failure to respond to these attempts results in classification as treatment abandonment. This recall system was implemented based on previous evidence showing improved retention (Scivoletto et al. 1992).

### 2.2 Sample

Participants' data were collected retrospectively, based on records from patients admitted between 1996 and 2023. Inclusion criteria to PROMUD are: being 18 years or older, having a DSM‐IV or DSM‐5 diagnosis of Substance Dependence, residing in the Greater São Paulo area and consented to participate. Exclusion criteria were: severe clinical or psychiatric issues from alcohol or drug use (such as delirium tremens, psychosis), intellectual impairment as categorised under DSM‐IV (Mental Retardation/Dementia) or DSM‐5 (Intellectual Disability).

Patients were referred to the outpatient clinic through Brazil's public healthcare system (Sistema Único de Saúde—SUS), by private psychiatrists, or spontaneously via the institution's website. All patients underwent an initial screening conducted by a psychiatrist, during which basic sociodemographic and clinical information was collected to determine whether they would start treatment at the clinic and thus be eligible for inclusion in the study. We divided our sample into two distinct populations: (1) pretreatment dropout group: women who were screened and accepted but never returned for the first treatment visit, and (2) treatment group: women who were screened and initiated treatment following their first outpatient appointment. For this study, we excluded only participants for whom data on treatment duration or the reason for treatment discontinuation were unavailable.

### 2.3 Variables

Sociodemographic data characteristics and other exposures were collected at the moment of screening. The variables collected included age at admission defined as the patient's age (in years) at the time of the initial screening visit, race/ethnicity (categorised as white, black, and other), education level (categorised as incomplete primary school, complete primary school, complete middle school), occupational status (categorised as retired, employed, housewife, unemployed), marital status (has a partner, as either having a partner—married or in a stable union—or without a partner—single, divorced, or widowed—) and income level (defined as low—1 minimum wage salary or less—and high—more than one minimum wage salary). The main substance of use was defined by a psychiatrist after the first interview at screening and was categorised as alcohol, cannabinoids, cocaine/crack, and other. The variable year at admission is a numerical variable that indicates the year in which the patient began treatment.

A comprehensive questionnaire was applied at the first appointment and during outpatient care. The variables collected included motherhood status (has children, with or without children), sexual orientation (heterosexual, homosexual or bisexual), previous suicide attempt, main reason for seeking treatment (because of self/other person), and whether the participant has ever experienced sexual violence.

Dropout was defined as leaving treatment for reasons other than referral, discharge due to clinical improvement, administrative discharge, or death. Hospitalisation was not classified as dropout, as patients admitted to the inpatient unit—managed by a separate clinical team within the same hospital—continued their care during hospitalisation and resumed outpatient follow‐up after discharge. Time to dropout was calculated as the number of weeks in treatment from the first appointment until dropout. Discontinued treatment due to referral to another service, discharge following clinical improvement, administrative discharge, or death were censored in the survival analyses.

### 2.4 Statistical Analyses

Analyses were conducted using R 4.2 and packages *Survival* and *Amelia*. The baseline characteristics of patients were compared using χ^2^ statistics for categorical variables and *t*‐tests for continuous variables. A *p* value of less than 0.05 was considered statistically significant. *P* values for the Fisher's exact tests were estimated with 10 000 Monte Carlo samples. We performed descriptive statistics for both the pretreatment dropout group and treatment group.

For our first aim, we included all participants, and we fitted a logistic regression with the binary outcome pretreatment dropout. Predictors were age strata, primary substance of choice, education level, and race. Estimates for Odds Ratios (ORs) were obtained, with statistical significance evaluated using Wald tests.

For our second aim we included only the treatment group to compare treatment retention across the three age categories. Survival curves were estimated using the Kaplan–Meier method, log‐hazard‐based 95% confidence intervals were constructed and a log‐rank test was performed to evaluate differences amongst the curves. The primary outcome was the time to event, measured in weeks, with a maximum treatment period of 104 weeks. The event was defined as dropout from treatment.

**FIGURE 1:**
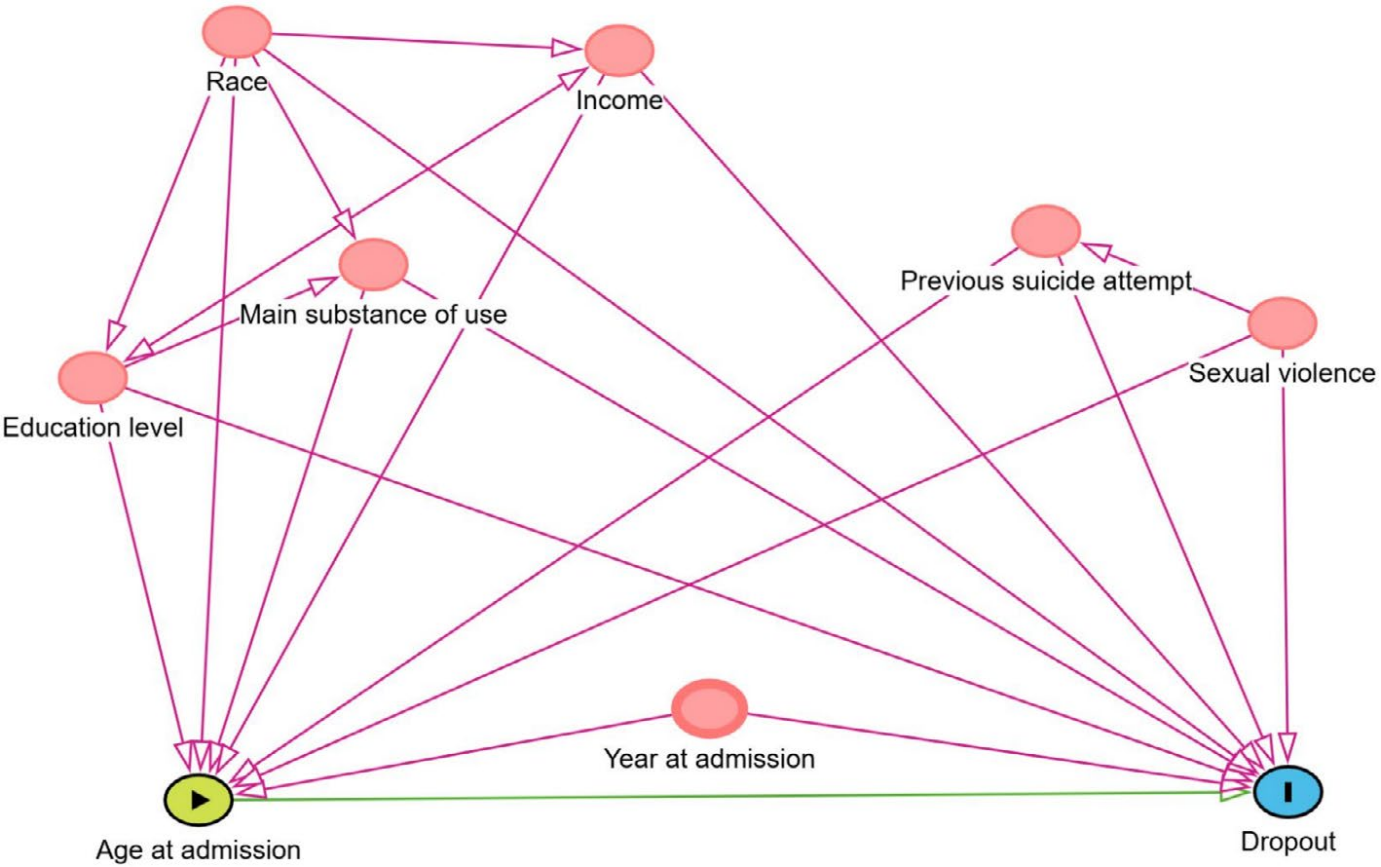
Directed acyclic graph (DAG) depicting the epidemiological model for the association between age at admission and dropout. Variables in red are ancestral variables of both exposure and outcome, and thus are recognised as confounders in the theoretical model.

For our third aim, a Cox proportional hazards model was fitted to assess the association between covariates and time to event. Age stratum was the main exposure variable, the chosen outcome was time to dropout from outpatient treatment. Additional variables, recognised as potential confounders, were also included. These confounders were identified using a theoretical framework constructed using Direct Acyclic Graph Methodology (DAG) from the dagitty software (Textor et al. 2016) (Figure 1). The identified confounders were: race, year at admission, previous suicide attempt, history of sexual violence, income, education level, main substance of use. As the Schoenfeld residuals suggested nonproportional hazards for year at admission and main substance of use: cocaine/crack and others, we refit the Cox model with linear time‐varying effects on these covariates. Hazard ratios (HRs) were then estimated for each covariate, with statistical significance evaluated using Wald tests.

As some patients did not have complete covariate data, we employed multiple imputation to address missing data. We generated 20 imputed datasets using the predictive mean matching (PMM) algorithm in mice (Van Buuren and Groothuis‐Oudshoorn 2011). For both the logistic regression and Cox proportional hazards model, which requires complete datasets, we repeated the analysis across these imputed pseudo‐datasets. The final results were obtained by pooling the parameter estimates from each imputed dataset, following Rubin's Rule (Rubin 1996).

## 3 Results

Out of 850 eligible participants, 756 were included in the study, with 94 excluded due to missing information on treatment duration or reasons for dropout.

The pretreatment dropout group included 120 participants: 31 emerging adults (EAs, 18–25), 85 middle‐aged adults (MAAs, 26–59), and 4 older adults (OAs, 60+). White participants represented 39.2% followed by black (15.8%). Low education levels were predominant. Most women had either incomplete (15.8%) or complete primary education (12.5%), whilst a smaller proportion had completed secondary (10.8%) or tertiary education (2.5%). No statistically significant differences in race and education level were found across age groups. 27.5% of participants did not have a partner. EAs primarily used cocaine/crack (71.0%), whilst alcohol was predominant in MAAs (51.8%) and OAs (75.0%) (Table 1).

**TABLE 1:** Characteristics of the sample.

Pretreatment dropout	Emerging adults (*N* = 31)	Middle aged adults (*N* = 85)	Older adults (*N* = 4)	Total (*N* = 120)	*p*
Race					
White	12 (38.7%)	34 (40.0%)	1 (25.0%)	47 (39.2%)	0.797
Black	8 (25.8%)	11 (12.9%)	0 (0%)	19 (15.8%)	
Asian or Indigenous	0 (0%)	1 (1.2%)	0 (0%)	1 (0.8%)	
Missing	11 (35.5%)	39 (45.9%)	3 (75.0%)	53 (44.2%)	
Education level					
Incomplete primary	6 (19.4%)	13 (15.3%)	0 (0%)	19 (15.8%)	0.59
Complete primary	7 (22.6%)	7 (8.2%)	1 (25.0%)	15 (12.5%)	
Complete secondary	2 (6.5%)	11 (12.9%)	0 (0%)	13 (10.8%)	
Complete tertiary	0 (0%)	3 (3.5%)	0 (0%)	3 (2.5%)	
Missing	16 (51.6%)	51 (60.0%)	3 (75.0%)	70 (58.3%)	
Has partner					
Yes	2 (6.5%)	11 (12.9%)	0 (0%)	13 (10.8%)	0.727
No	10 (32.3%)	22 (25.9%)	1 (25.0%)	33 (27.5%)	
Missing	19 (61.3%)	52 (61.2%)	3 (75.0%)	74 (61.7%)	
Main substance					
Alcohol	5 (16.1%)	44 (51.8%)	3 (75.0%)	52 (43.3%)	< 0.001
Cannabinoids	4 (12.9%)	2 (2.4%)	1 (25.0%)	7 (5.8%)	
Cocaine and crack	22 (71.0%)	25 (29.4%)	0 (0%)	47 (39.2%)	
Other	0 (0%)	7 (8.2%)	0 (0%)	7 (5.8%)	
Missing	0 (0%)	7 (8.2%)	0 (0%)	7 (5.8%)	

Treatment	Emerging adults (*N* = 94)	Middle aged adults (*N* = 500)	Older adults (*N* = 42)	Total (*N* = 636)	*p*
Race					
White	64 (68.1%)	338 (67.6%)	30 (71.4%)	432 (67.9%)	0.992
Black	21 (22.3%)	119 (23.8%)	8 (19.0%)	148 (23.3%)	
Asian or Indigenous	1 (1.1%)	5 (1.0%)	0 (0%)	6 (0.9%)	
Missing	8 (8.5%)	38 (7.6%)	4 (9.5%)	50 (7.9%)	
Occupational status					
Unemployed	54 (57.4%)	177 (35.4%)	5 (11.9%)	236 (37.1%)	< 0.001
Housewife	1 (1.1%)	36 (7.2%)	9 (21.4%)	46 (7.2%)	
Employed	21 (18.1%)	171 (30.6%)	4 (10.0%)	196 (27.5%)	
Retired	1 (1.1%)	23 (4.6%)	15 (35.7%)	39 (6.1%)	
Missing	18 (19.1%)	87 (17.4%)	9 (21.4%)	114 (17.9%)	
Education level					
Incomplete primary	16 (17.0%)	97 (19.4%)	14 (33.3%)	127 (20.0%)	< 0.001
Complete primary	29 (30.9%)	59 (11.8%)	5 (11.9%)	93 (14.6%)	
Complete secondary	35 (37.2%)	188 (37.6%)	12 (28.6%)	235 (36.9%)	
Complete tertiary	5 (5.3%)	98 (19.6%)	7 (16.7%)	110 (17.3%)	
Missing	9 (9.6%)	58 (11.6%)	4 (9.5%)	71 (11.2%)	
Income					
Low	20 (21.3%)	144 (28.8%)	10 (23.8%)	174 (27.4%)	0.212
High	35 (37.2%)	134 (26.8%)	12 (28.6%)	181 (28.5%)	
Missing	39 (41.5%)	222 (44.4%)	20 (47.6%)	281 (44.2%)	
Has children					
No	36 (38.3%)	79 (15.8%)	4 (9.5%)	119 (18.7%)	< 0.001
Yes	18 (19.1%)	267 (53.4%)	28 (66.7%)	313 (49.2%)	
Missing	40 (42.6%)	154 (30.8%)	10 (23.8%)	204 (32.1%)	
Sexual orientation					
Bisexual	15 (16.0%)	16 (3.2%)	1 (2.4%)	32 (5.0%)	0.0012
Heterosexual	49 (52.1%)	332 (66.4%)	33 (78.6%)	414 (65.1%)	
Homosexual	7 (7.4%)	31 (6.2%)	1 (2.4%)	39 (6.1%)	
Other	0 (0%)	1 (0.2%)	0 (0%)	1 (0.2%)	
Missing	23 (24.5%)	120 (24.0%)	7 (16.7%)	150 (23.6%)	
Has partner					
Yes	18 (19.1%)	153 (30.6%)	11 (26.2%)	182 (28.6%)	0.133
No	64 (68.1%)	284 (56.8%)	26 (61.9%)	374 (58.8%)	
Missing	12 (12.8%)	63 (12.6%)	5 (11.9%)	80 (12.6%)	
Previous suicide attempt					
No	23 (24.5%)	166 (33.2%)	20 (47.6%)	209 (32.9%)	0.00465
Yes	35 (37.2%)	115 (23.0%)	5 (11.9%)	155 (24.4%)	
Missing	36 (38.3%)	219 (43.8%)	17 (40.5%)	272 (42.8%)	
Main substance					
Alcohol	20 (21.3%)	306 (61.2%)	36 (85.7%)	362 (56.9%)	< 0.001
Cannabinoids	10 (10.6%)	9 (1.8%)	0 (0%)	19 (3.0%)	
Cocaine and crack	51 (54.3%)	138 (27.6%)	3 (7.1%)	192 (30.2%)	
Other	10 (10.6%)	40 (8.0%)	2 (4.8%)	52 (8.2%)	
Missing	3 (3.2%)	7 (1.4%)	1 (2.4%)	11 (1.7%)	
Main reason help seeking					
Other person	40 (42.6%)	153 (30.6%)	22 (52.4%)	215 (33.8%)	0.0112
Self	31 (33.0%)	224 (44.8%)	13 (31.0%)	268 (42.1%)	
Missing	23 (24.5%)	123 (24.6%)	7 (16.7%)	153 (24.1%)	
Sexual violence					
No	23 (24.5%)	175 (35.0%)	18 (42.9%)	216 (34.0%)	0.732
Yes	14 (14.9%)	75 (15.0%)	6 (14.3%)	95 (14.9%)	
Missing	57 (60.6%)	250 (50.0%)	18 (42.9%)	325 (51.1%)	

*Note:* Pretreatment dropout group includes participants who were screened and accepted but never returned for the first treatment visit, and Treatment group includes participants who were screened and initiated treatment following a first outpatient appointment. Associations between age group and other demographic and clinical variables were analysed using Fisher's exact tests.

The treatment group included 94 EAs, 500MAAs, and 42 OAs. Most participants were white (67.9%), followed by black (23.3%), with no significant racial differences across age groups (Table 1).

The 37.1% of participants were unemployed, particularly EAs (57.4%). EAs also showed similar rates of primary (30.9%) and secondary (37.2%) education completion. In contrast, MAAs had the highest rate of tertiary education completion (19.6%), whilst 33.3% of OAs had not completed primary education. These differences were statistically significant (*p* < 0.001).

The 49.2% of participants had children. EAs had a higher rate of bisexuality (16.0%) compared to MAAs (3.2%) and OAs (2.4%). More than half (58.8%) did not have a partner. 37.2% of EAs reported suicide attempts, compared to 23% of MAAs and 11.9% of OAs. EAs primarily used cocaine/crack (54.3%), whilst alcohol was predominant in MAAs and OAs, with significant substance use differences across age groups. There was no significant difference in the history of sexual violence and income.

The logistic regression analysis for the outcome pretreatment dropout revealed that MAAs had significantly lower odds of missing the initial appointment compared to EAs, with an odds ratio of 0.56 (*p* = 0.025) (Table 2).

**TABLE 2:** Logistic regression results for pretreatment dropout.

Variable		Coefficient	SE	OR	Statistic	df	*p*
Year at admission		0.01	0.01	1.01	0.45	590.8	0.653
Age stratum	Emerging adults	ref					
	Middle aged adults	−0.58	0.26	0.56	−2.24	755.1	**0.025**
	Older adults	−1.13	0.59	0.32	−1.92	769.8	0.055
Main substance	Alcohol	ref					
	Cannabinoids	0.43	0.50	1.54	0.86	726.8	0.389
	Cocaine/Crack	0.26	0.24	1.30	1.08	614.8	0.282
	Others	−0.03	0.40	0.97	−0.08	640.2	0.936
Education level	Incomplete primary	ref					
	Complete primary	0.16	0.36	1.18	0.45	233.3	0.653
	Completed secondary	−0.20	0.35	0.82	−0.58	175.0	0.562
	Completed tertiary	−0.22	0.44	0.80	−0.51	137.7	0.611
Race	White	ref					
	Indigenous or Asian	0.04	1.14	1.04	0.03	175.7	0.974
	Black	−0.03	0.29	0.97	−0.10	136.8	0.918

*Note:* Twenty imputed datasets using the predictive mean matching (PMM) method. Estimates were combined according to Rubin's rules. Statistically significant results (*p* < 0.05) are shown in **bold**.

Abbreviations: CI, confidence interval; OR, odds ratio.

**FIGURE 2:**
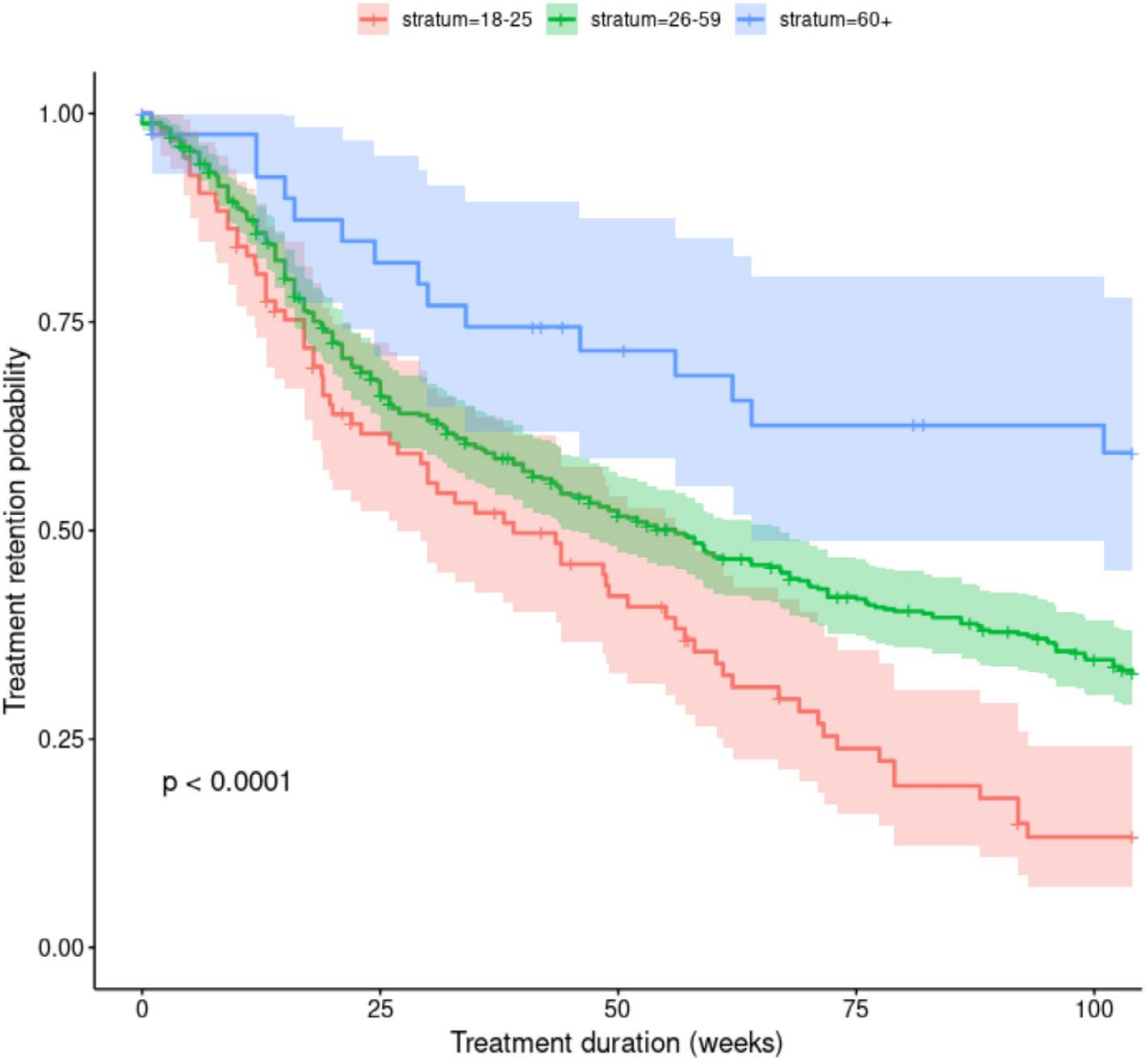
Retention probability KM‐based estimates and Greenwood 95% CI for each stratum at times 25, 52, and 104.

The Kaplan Meier curves were estimated (Figure 2). EAs had the lowest retention, with 62% at 25 weeks, 41% at 52 weeks, and only 13% at 104 weeks. MAAs showed moderate retention (66%, 51%, and 33%). OAs had higher retention (82%, 72%, and 59%). Log rank test *p* < 0.0001 (Table 3).

**TABLE 3:** Retention probability.

Time (weeks)	Retention probability		
	18–25 years	26–59 years	60+ years
25	62%	66%	82%
52	41%	51%	72%
104	13%	33%	59%

*Note:* Retention probability for age strata at times 25, 52 and 104 weeks.

Table 4 illustrates the results from the Cox proportional hazards model. Compared to EAs, the MAAs group has a lower dropout risk (HR = 0.67, *p* = 0.008), and OAs have an even lower risk (HR = 0.30, *p* = 0.000). Having completed tertiary education represented lower risk of dropout compared to incomplete primary school (HR = 0.65, *p* = 0.038).

**TABLE 4:** Cox regression model with time interactions (*x* * *t*): Final estimates.

Variable		Coefficient	SE	HR	Statistic	df	*p*
Age stratum	Emerging adults	ref					
	Middle aged adults	−0.40	0.15	0.67	−2.68	364.5	**0.008**
	Older adults	−1.20	0.30	0.30	−3.96	366.4	**0.000**
Main substance	Alcohol	ref					
	Cannabinoids	−0.04	0.33	0.96	−0.11	362.3	0.912
	Cocaine/Crack	0.45	0.19	1.57	2.41	353.8	**0.017**
	Time trend: Cocaine/crack use	−0.01	0.00	0.99	−2.06	359.2	**0.041**
	Others	−0.89	0.38	0.41	−2.35	347.3	**0.020**
	Time trend: Others	0.02	0.01	1.02	2.71	357.0	**0.007**
Education level	Incomplete primary	ref					
	Complete primary	−0.07	0.18	0.93	−0.41	298.7	0.681
	Complete secondary	−0.21	0.15	0.81	−1.39	324.8	0.166
	Completed tertiary	−0.43	0.20	0.65	−2.08	278.0	**0.038**
Race	White	ref					
	Indigenous or Asian	−0.62	0.72	0.54	−0.86	337.6	0.392
	Black	−0.21	0.13	0.81	−1.61	331.0	0.108
Year at admission	Time trend: Year at admission	0.43	0.11	1.54	3.89	340.7	**0.000**
		−0.01	0.00	0.99	−3.25	373.3	**0.001**
Previous suicide attempt	No	ref					
	Yes	−0.09	0.13	0.92	−0.68	162.1	0.500
Income	Low	ref					
	High	−0.05	0.13	0.95	−0.41	161.0	0.681
Sexual abuse	No						
	Yes	−0.12	0.14	0.89	−0.86	183.0	0.392

*Note:* Statistically significant results (*p* < 0.05) are shown in **bold**.

Abbreviations: CI, confidence interval; df, degrees of freedom; HR, hazard ratio; SE, standard error; Stat, statistic.

**FIGURE 3:**
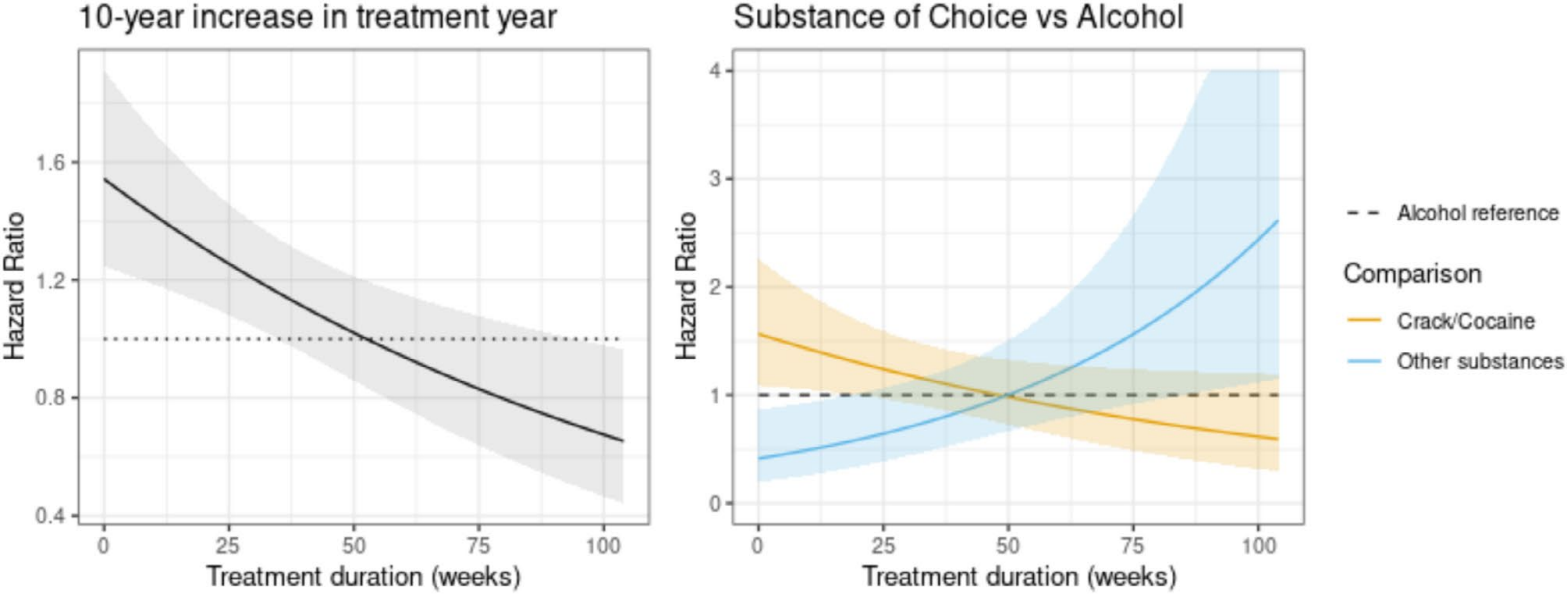
Treatment duration effects: Time‐Varying Hazard Ratios Over Time. Hazard ratios for risk of dropout. Hazard ratios (HR) were estimated from the Cox regression model, with 95% confidence intervals calculated using the delta method.

Substance‐specific differences in dropout risk were observed across the treatment timeline. Compared to alcohol users, cocaine/crack users displayed a significantly elevated risk of dropout (HR = 1.5, *p* < 0.05) during the first months of treatment. Conversely, users of other substances demonstrated a biphasic pattern: a reduced dropout risk in the initial phase (HR = 0.5) followed by an increase near treatment completion (HR = 2.5, *p* < 0.05). Time‐varying covariate analysis further revealed a progressive rise in dropout risk over the first 6 months of treatment (HR = 1.4, *p* < 0.05) per 10‐year increment in treatment year. These associations are visually synthesised in Figure 3. Retention rates were not significantly influenced by previous suicide attempt, known sex violence history or income.

## 4 Discussion

For our first aim, we studied predictors of pretreatment dropout in our sample and found that MAAs are more likely to come to the first treatment appointment when compared to EAs. The findings regarding OAs were not significant, probably due to the smaller size of this subsample.

For our second aim, we compared SUD treatment retention across three age strata. According to our initial hypothesis, EAs exhibited lower treatment retention compared to the other age strata (EAs 13% MAAs 33% OAs 59%). These findings are consistent with previous research indicating that EAs have higher dropout rates in outpatient programmes for substance use disorders. Studies also indicate that whilst EAs may experience rapid disengagement early in treatment, OAs tend to show better retention, possibly due to greater life experience and a more stable psychosocial environment (Bourion‐Bédès et al. 2020; Lappan et al. 2020).

Current research emphasises the necessity of recognising the distinct treatment requirements of EAs with SUD, which may involve the need for intensified therapeutic interventions (Andersson et al. 2021; Dalton et al. 2021; DiClemente et al. 2009; Satre et al. 2003, 2004; Schuman‐Olivier et al. 2014; Smith et al. 2010, 2011; Welsh et al. 2019). If 90% of patients in this age group leave treatment, then retention strategies must become a priority for intervention.

For our third aim, our findings suggest that higher educational level is a protective factor amongst women, specifically those with a completed tertiary education. This finding is in line with other studies, which identified low educational attainment as a significant risk factor for treatment dropout (Brorson et al. 2013; Lappan et al. 2020). However, it is important to note that studies focused on emerging adult populations have not consistently established this association, and existing literature lacks sufficient evidence addressing this relationship specifically in women (McHugh et al. 2018; Greenfield et al. 2007). It is presumed that social stigma constitutes one of the primary barriers to treatment access for women who use substances. In this context, low educational attainment may exacerbate the situation, contributing to a higher risk of treatment dropout compared to men. Women with lower education levels may face additional challenges, such as a lack of resources and social support, which can hinder their adherence to treatment programmes. These findings underscore the importance of considering educational and social factors when developing targeted interventions for women.

In addition to differences between age groups, our analysis with time‐dependent covariates revealed a progressive rise in dropout risk over the first 6 months of treatment, especially in patients admitted in more recent years. Specifically, for each 10‐year increment in the year at admission into treatment, a significant 40% increase in the risk of dropout was observed. This finding may reflect structural changes in the health system or sociocultural transformations over time, such as increased precariousness of work, difficulties in continuous access to the service, or changes in the profile of patients. It is also possible that engagement in continuing care has become more challenging in recent contexts, which highlights the need to adapt treatment models to new social and institutional realities (Bourion‐Bédès et al. 2020; Lappan et al. 2020; Andersson et al. 2021; Rhodes et al. 2018).

Our findings suggest that women who use cocaine and crack are more likely to drop out during the first months of treatment. The literature presents controversial findings on the association between primary substance use and the risk of abandoning treatment for substance dependence; a systematic review of 124 studies found 19 that explored the connection between cocaine use and treatment dropout (Brorson et al. 2013). Of these, three studies indicated that cocaine users are more likely to drop out of addiction treatment. Although some studies have addressed the relationship between substance type and treatment adherence, few have examined this dynamic in EAs. The lack of clear data highlights the need for additional research to explore the characteristics of this age group concerning the use of cocaine and crack (Brorson et al. 2013; Greenfield et al. 2010; Lappan et al. 2020; McCaul et al. 2001).

Our findings bring important insights related not only to the quantity but also the chronology of dropouts in female EAs. These insights can be translated into changes in clinical practises when managing this population. We hereby propose that emerging adulthood be recognised as a distinct clinical subgroup amongst women, requiring immediate and continuous tailored interventions. We suggest implementing early‐stage risk stratification and intervention protocols for this population, which can improve treatment retention and alter the course of the disease.

The Literature highlights several effective integrated strategies to increase treatment retention—such as behavioural therapies, medication‐assisted treatment, low‐threshold treatment models, and process improvement initiatives like the NIATx model (Network for the Improvement of Addiction Treatment)—which could be crucial for altering the trajectory of SUD amongst EAs women, promoting sustained engagement and improved long‐term outcomes (Dalton et al. 2021; Hadland et al. 2018; Kennedy et al. 2021; Wakeman et al. 2022; Hoffman et al. 2008).

### 4.1 Strengths

To our knowledge, this is the largest clinical sample studying treatment retention of female patients in Brazil. Our clinical sample has been studied since 1996, a time when gender inclusion in clinical trials was still developing globally (Bae 2022). Our unique focus on the female population offers important insights into a population underrepresented in the context of low‐ and middle‐income countries (LMIC), contributing to the understanding of gender differences in treatment retention for treatment of SUDs. It also allows for generalisation of findings from high‐income countries (HIC).

### 4.2 Limitations

The external validity of our findings is impaired by the sociodemographic peculiarities of our sample, which is mostly white and has a higher education level and income than the Brazilian average. Also, our treatment service is located in a tertiary hospital in the center of São Paulo and requires weekly outpatient appointments.

Most Brazilians diagnosed with SUD are treated at CAPS AD, which are community‐based primary care services. Therefore, our data do not reflect the national standard of mental health care and are not generalizable to the population served by CAPS AD, which is predominantly male and characterised by lower educational attainment and income levels. On the other hand, our sample sheds light on women with SUD, an underrepresented population which faces more barriers to access treatment. Moreover, our sample size for EAs is not large enough to perform in depth analyses.

## 5 Conclusions

Emerging adulthood is a critical period for health interventions, particularly in the context of SUD. The gender gap is narrowing, and women are still underrepresented in studies. We found very high pretreatment and treatment dropout rates amongst emerging adults in our sample. These findings underscore the necessity for early‐stage risk stratification and tailored intervention protocols for this population.

## Funding

The authors have nothing to report.

## Ethics Statement

The authors assert that all procedures contributing to this work comply with the ethical standards of the relevant national and institutional committees on human experimentation and with the Helsinki Declaration of 1975, as revised in 2008. All participants consented to participate. This study was approved by the Research Ethics Committee of the Hospital das Clínicas da Faculdade de Medicina da Universidade de São Paulo (HCFM/USP), through the Plataforma Brasil system (Certificate of Presentation for Ethical Review—CAAE: 71050623.8.0000.0068), under approval number 6.185.217.

## Conflicts of Interest

The authors declare no conflicts of interest.

## Supporting information


**Data S1:** Supporting Information.

**Figure S1:** Schoenfeld residual plots assessing the proportional hazards assumption for covariates in the Cox model (treatment group).

## Data Availability

The data that support the findings of this study are available from the corresponding author upon reasonable request.
