# Supplementary material for: Gone Too Soon: Higher Pretreatment and Treatment Dropout Among Emerging Adults in a Women‐Specific Outpatient Treatment Service for Substance Use Disorders in Brazil
Source: Early Interv Psychiatry. 2026 Jan 11;20(1):e70133. doi: 10.1111/eip.70133 (PMC12791191; doi:10.1111/eip.70133)
Supplement: Supplementary file 1 — Data S1: Supporting Information. [file EIP-20-0-s001.docx]

**Statistical Analysis Codes**

The following sections contain the R code used to perform the statistical analyses described in the manuscript. This supplementary material includes all steps used in the statistical analysis, including data preprocessing, descriptive statistics, and survival analysis.

*Table 1*

################# Table 1: tratamento e triagem ###########################
 # Load the packages
 library(dplyr)
 library(survival)
 library(ggplot2)
 library(table1)

 df = read.csv('dados_final/dados_consolidados_t0.csv')

 dat_esa = readxl::read_excel(
 'early_interventions/ESA PROMUD early intervention 10 10.xlsx')

 # View(dat)

 # Keep only the first occurrence of each repeated id_promud
 dat_esa_clean <- dat_esa %>%
 distinct(id_promud, .keep_all = TRUE)

 # Perform the left join based on the id_promud column
 df <- df %>%
 left_join(dat_esa_clean, by = "id_promud")

 ###### prepare columns

 # preparar covariaveis
 df$event <- df$status == 'abandono'

 # n inicial = 854
 nrow(df)

 # com indicador de evento = 790 (o resto não tem status)
 df = df[!is.na(df$event),]
 nrow(df)

 df$duracao_tratamento_weeks = df$duracao_tratamento/7

 # tirando quem abandonou, mas não tem data de abandono = 774
 df = df[!(is.na(df$duracao_tratamento_weeks) & df$event),]
 nrow(df)

 # colocando duracao tratamento de quem ta em tratamento
 # = 10-06-24 - entrada
 df$duracao_tratamento_weeks[df$status == 'em tratamento'] =
 (as.numeric(as.Date('2024-06-10') -
 as.Date(df$sociodem_data_t0[df$status == 'em tratamento'])))/7

 # varias altas sem data, n = 761
 df[df$status=='alta médica'&is.na(df$duracao_tratamento_weeks),]$id_promud
 df = df[!is.na(df$duracao_tratamento_weeks),]
 nrow(df)

 # janela de dois anos
 df$event[which(df$duracao_tratamento_weeks>104)] = F
 df$duracao_tratamento_weeks[df$duracao_tratamento_weeks>104] = 104

 # TODO: ver qual o problema com esses tratamentos
 # que tem duracao negativa
 # 3 com duracao de tratamento negativa, n= 758
 df = df[-which(df$duracao_tratamento_weeks <0),]
 nrow(df)

 # faixa etaria
 df$faixa_idade = ifelse(
 df$sociodem_idade_t0 < 26, "18-25",
 ifelse(
 df$sociodem_idade_t0 <= 59, "26-59",
 ifelse(
 df$sociodem_idade_t0 >= 60, "60+",
 NA
 )
 )
 )

 df$faixa_idade[is.na(df$faixa_idade)] = 'Missing'

 df$ano_t0 = as.numeric(substr(df$sociodem_data_t0, 0, 4))

 # mais que um salario minimo nacional
 df$faixa_renda_t0 = ifelse(df$sociodem_renda_mensal_t0>1412, '<1 salário',
 '>1 salário')

 table(df$sociodem_escolaridade_t0)

 tmp = tribble(
 ~sociodem_escolaridade_t0, ~escolaridade,
 'analfabeta', 'incomplete primary',
 'fundamental 1 incompleto', 'incomplete primary',
 'fundamental 1 completo', 'incomplete primary',
 'fundamental 2 incompleto', 'incomplete primary',
 'fundamental 2 completo', 'complete primary',
 'médio incompleto', 'complete primary',
 'médio completo', 'complete secondary',
 'superior incompleto', 'complete secondary',
 'superior completo', 'complete tertiary'
 )

 df = left_join(df, tmp)

 table(df$sociodem_raca)

 tmp = tribble(
 ~sociodem_raca, ~raca,
 'branca', 'branca',
 'preta', 'pretaparda',
 'parda', 'pretaparda',
 'amarela', 'amarela',
 'indigena', 'indigena'
 )

 df = left_join(df, tmp)


 table(df$dependencia_principal)

 tmp = tribble(
 ~dependencia_principal, ~substancia,
 'alcool', 'alcool',
 'canabinoides', 'canabinoides',
 'cocaina', 'cocainacrack',
 'crack', 'cocainacrack',
 'opioides', 'outras',
 'outras', 'outras',
 'sedativos', 'outras'
 )

 df = left_join(df, tmp)

 ##SEMANAS##
 df$status <- ifelse(df$duracao_tratamento_weeks == 104, "+2 anos", df$status)

 df$filho <- ifelse(df$sociodem_num_filhos_t0 >=1,
 "sim", "não")

 df$psi_score = (df$psi_depressao_30d_t0 =='sim') + (df$psi_ansiedade_30d_t0=='sim') + (df$psi_dificuldade_dormir_30d_t0=='sim')

 ####### ESA SOMA ################

 df$esa_soma <- df$consumo_t0 + df$ocupacao_t0 + df$lazer_t0 + df$familia_t0

 pvalue <- function(x, ...) {
 # Extract the values and groups (strata)
 y <- unlist(x)
 g <- factor(rep(1:length(x), times=sapply(x, length)))

 if (is.numeric(y)) {
 # For numeric variables, perform ANOVA (as there are >2 groups)
 p <- summary(aov(y ~ g))[[1]]["Pr(>F)"][1]
 } else {
 # For categorical variables, perform a chi-squared test of independence
 p <- fisher.test(table(y, g), workspace = 1e8, simulate.p.value = T, B= 1e5)$p.value
 }
 # Format the p-value
 c("", format.pval(p, digits = 3, eps = 0.001))
 }

 label(df$substancia) <- "Primary Dependency"
 label(df$raca) <- "Race"
 label(df$faixa_idade) <- "Age Category"

 df$status2 = ifelse(df$status == 'abandono na triagem', 'abandono na triagem: sim',
 'abandono na triagem: nao')

 table1(~ raca + escolaridade + sociodem_estadocivil_t0 + substancia | faixa_idade,
 data = df[df$status2 == 'abandono na triagem: sim' &
 df$faixa_idade != 'Missing',],
 # To avoid overall statistics interfering with the p-value function
 overall = T,
 extra.col = list(`P-value` = pvalue)
 )
 table1(~ raca + sociodem_sitprofissional_t0 + escolaridade + faixa_renda_t0 +
 filho + sociodem_orientsexual + sociodem_estadocivil_t0 +
 psi_tentativa_suicidio_vida_t0 + substancia +
 motivo_tratamento + abuso_sex_conhecido_t0 | faixa_idade,
 data = df[df$status2 == 'abandono na triagem: nao' &
 df$faixa_idade != 'Missing',],
 # To avoid overall statistics interfering with the p-value function
 overall = T,
 extra.col = list(`P-value` = pvalue)
 )

*Tables 2 and 3*

#####
 #####
 #####
 library(gamlss)
 library(mice)

 df <- read.csv('dados_final/dados_consolidados_t0.csv')

 # n inicial = 854
 nrow(df)

 # com indicador de evento = 790 (o resto nÃ£o tem status)
 df = df[!is.na(df$status),]
 nrow(df)

 # y = 1 se faltou no abandonou logo na triagem
 df$y = df$status == 'abandono na triagem'

 ####### criar variaveis

 df$ano_t0 = as.numeric(substr(df$sociodem_data_t0, 0, 4))

 ###### criar covariaveis

 # isso aqui Ã© sÃ³ pra usar dps
 tmp = tribble(
 ~dependencia_principal, ~substancia,
 'alcool', 'alcool',
 'canabinoides', 'canabinoides',
 'cocaina', 'cocainacrack',
 'crack', 'cocainacrack',
 'opioides', 'outras',
 'outras', 'outras',
 'sedativos', 'outras'
 )

 tmp2 = tribble(
 ~sociodem_escolaridade_t0, ~escolaridade,
 'analfabeta', 'incomplete primary',
 'fundamental 1 incompleto', 'incomplete primary',
 'fundamental 1 completo', 'incomplete primary',
 'fundamental 2 incompleto', 'incomplete primary',
 'fundamental 2 completo', 'complete primary',
 'mÃ©dio incompleto', 'complete primary',
 'mÃ©dio completo', 'complete secondary',
 'superior incompleto', 'complete secondary',
 'superior completo', 'complete tertiary'
 )


 tmp3 = tribble(
 ~sociodem_raca, ~raca,
 'branca', 'branca',
 'preta', 'pretaparda',
 'parda', 'pretaparda',
 'amarela', 'indigenaamarela',
 'indigena', 'indigenaamarela'
 )


 colMeans(is.na(df[df$y,])) # % missing em cada coluna entre os que faltaram caso novo

 # essas fazem sentido usar na triagem
 covariates = df %>% select(ano_t0, sociodem_idade_t0,
 dependencia_principal, sociodem_raca, sociodem_escolaridade_t0)

 covariates[] <- lapply(covariates, function(x) {
 if (is.character(x)) as.factor(x) else x
 })

 ###### imputacoes
 num_imp = 20
 covariates_imp = mice(covariates, m = num_imp,
 method = 'pmm', print = F, seed = 517207)


 results <- list()

 for (i in 1:num_imp) {
 covariates_imp1 = complete(covariates_imp, i)

 ###### transformar variaveis

 # faixa etaria
 covariates_imp1$faixa_idade = ifelse(
 covariates_imp1$sociodem_idade_t0 < 26, "18-25",
 ifelse(
 covariates_imp1$sociodem_idade_t0 <= 59, "26-59",
 ifelse(
 covariates_imp1$sociodem_idade_t0 >= 60, "60+",
 NA
 )
 )
 )
 covariates_imp1$sociodem_idade_t0 = NULL

 covariates_imp1 = left_join(covariates_imp1, tmp,
 by = 'dependencia_principal')
 covariates_imp1$dependencia_principal = NULL

 covariates_imp1 = left_join(covariates_imp1, tmp2,
 by = 'sociodem_escolaridade_t0')
 covariates_imp1$sociodem_escolaridade_t0 = NULL
 covariates_imp1$escolaridade = factor(covariates_imp1$escolaridade)
 covariates_imp1$escolaridade = relevel(
 covariates_imp1$escolaridade, ref = 'incomplete primary')
 covariates_imp1 = left_join(covariates_imp1, tmp3,
 by = 'sociodem_raca')
 covariates_imp1$sociodem_raca = NULL

 dat_fit = cbind(y = df$y,
 covariates_imp1)

 fit = glm(y ~ ., family = binomial, data = dat_fit)

 results[[i]] <- fit
 }

 result_table = pool(results)

 pooled = result_table$pooled

 data.frame(
 covariavel = pooled$term,
 coef = round(pooled$estimate,2),
 se = round(sqrt(pooled$t),2),
 OR = round(exp(pooled$estimate),2),
 stat = round(pooled$estimate/sqrt(pooled$t),2),
 df = round(pooled$df, 1),
 pval = round(2*pt(-abs(pooled$estimate/sqrt(pooled$t)), df=pooled$df),3)
 )

 # covariavel coef se OR stat df pval
 # 1 (Intercept) -13.94 28.14 0.00 -0.50 594.8 0.620
 # 2 ano_t0 0.01 0.01 1.01 0.45 590.8 0.653
 # 3 faixa_idade26-59 -0.58 0.26 0.56 -2.24 755.1 0.025
 # 4 faixa_idade60+ -1.13 0.59 0.32 -1.92 769.8 0.055
 # 5 substanciacanabinoides 0.43 0.50 1.54 0.86 726.8 0.389
 # 6 substanciacocainacrack 0.26 0.24 1.30 1.08 614.8 0.282
 # 7 substanciaoutras -0.03 0.40 0.97 -0.08 640.2 0.936
 # 8 escolaridadecomplete primary 0.16 0.36 1.18 0.45 233.3 0.653
 # 9 escolaridadecomplete secondary -0.20 0.35 0.82 -0.58 175.0 0.562
 # 10 escolaridadecomplete tertiary -0.22 0.44 0.80 -0.51 137.7 0.611
 # 11 racaindigenaamarela 0.04 1.14 1.04 0.03 175.7 0.974
 # 12 racapretaparda -0.03 0.29 0.97 -0.10 136.8 0.918

*Table 4*

#####
 ##### AJUSTE COX COM IMPUTACAO PMM E INFERENCIA VIA REGRA DE RUBIN
 #####

 library(tidyverse)
 library(survival)
 library(Epi)
 library(popEpi)
 library(mgcv)
 library(mice)
 library(survminer)
 library(patchwork)

 df <- read.csv('dados_final/dados_consolidados_t0.csv')

 # preparar covariaveis
 df$event <- df$status == 'abandono'

 # n inicial = 854
 nrow(df)

 # com indicador de evento = 790 (o resto não tem status)
 df = df[!is.na(df$event),]
 nrow(df)

 # 667 que passaram da triagem
 df = df[-which(df$status == 'abandono na triagem'),]
 nrow(df)

 df$duracao_tratamento_weeks = df$duracao_tratamento/7

 # tirando quem abandonou, mas não tem data de abandono = 651
 df = df[!(is.na(df$duracao_tratamento_weeks) & df$event),]
 nrow(df)

 # colocando duracao tratamento de quem ta em tratamento
 # = 10-06-24 - entrada
 df$duracao_tratamento_weeks[df$status == 'em tratamento'] =
 (as.numeric(as.Date('2024-06-10') -
 as.Date(df$sociodem_data_t0[df$status == 'em tratamento'])))/7

 # varias altas sem data, n = 641
 df[df$status=='alta médica'&is.na(df$duracao_tratamento_weeks),]$id_promud
 df = df[!is.na(df$duracao_tratamento_weeks),]
 nrow(df)

 # janela de dois anos
 df$event[which(df$duracao_tratamento_weeks>104)] = F
 df$duracao_tratamento_weeks[df$duracao_tratamento_weeks>104] = 104

 # TODO: ver qual o problema com esses tratamentos
 # que tem duracao negativa
 # 3 com duracao de tratamento negativa, n= 638
 df = df[-which(df$duracao_tratamento_weeks <0),]
 nrow(df)

 # faixa etaria
 df$faixa_idade = ifelse(
 df$sociodem_idade_t0 < 26, "18-25",
 ifelse(
 df$sociodem_idade_t0 <= 59, "26-59",
 ifelse(
 df$sociodem_idade_t0 >= 60, "60+",
 NA
 )
 )
 )

 df$ano_t0 = as.numeric(substr(df$sociodem_data_t0, 0, 4))

 # mais que um salario minimo nacional
 df$faixa_renda_t0 = df$sociodem_renda_mensal_t0>1412

 table(df$sociodem_escolaridade_t0)

 tmp = tribble(
 ~sociodem_escolaridade_t0, ~escolaridade,
 'analfabeta', 'incomplete primary',
 'fundamental 1 incompleto', 'incomplete primary',
 'fundamental 1 completo', 'incomplete primary',
 'fundamental 2 incompleto', 'incomplete primary',
 'fundamental 2 completo', 'complete primary',
 'médio incompleto', 'complete primary',
 'médio completo', 'complete secondary',
 'superior incompleto', 'complete secondary',
 'superior completo', 'complete tertiary'
 )

 df = left_join(df, tmp)


 table(df$dependencia_principal)

 tmp = tribble(
 ~dependencia_principal, ~substancia,
 'alcool', 'alcool',
 'canabinoides', 'canabinoides',
 'cocaina', 'cocainacrack',
 'crack', 'cocainacrack',
 'opioides', 'outras',
 'outras', 'outras',
 'sedativos', 'outras'
 )

 df = left_join(df, tmp)

 table(df$sociodem_raca)


 tmp3 = tribble(
 ~sociodem_raca, ~raca,
 'branca', 'branca',
 'preta', 'pretaparda',
 'parda', 'pretaparda',
 'amarela', 'indigenaamarela',
 'indigena', 'indigenaamarela'
 )

 df = left_join(df, tmp3)


 covariates = df %>% dplyr::select(
 raca,
 substancia,
 faixa_renda_t0,
 escolaridade,
 ano_t0,
 psi_tentativa_suicidio_vida_t0,
 abuso_sex_conhecido_t0,
 # psi_dificuldade_dormir_30d_t0,
 # psi_depressao_30d_t0,
 # psi_ansiedade_30d_t0,
 faixa_idade
 )

 # remover unidades que, das 10 covariaveis, tem menos de 5, n = 480
 # to_drop = rowSums(is.na(covariates))>=6
 #
 # covariates = covariates[!to_drop,]
 # df = df[!to_drop,]
 #
 # nrow(df)

 ### transformar em fatores
 covariates[] <- lapply(covariates, function(x) {
 if (is.character(x)) as.factor(x) else x
 })

 covariates$escolaridade = relevel(
 covariates$escolaridade, ref='incomplete primary')

 # mice
 num_imp = 50
 covariates_imp = mice::mice(data = covariates,
 m=num_imp,
 method = 'pmm', print = F, seed = 13952854)

 results <- list()
 dat_fits <- list()

 for (i in 1:num_imp) {
 covariates_imp1 = complete(covariates_imp, i)

 # covariates_imp1$psi_comorbidade_score =
 # (covariates_imp1$psi_ansiedade_30d_t0=='sim') +
 # (covariates_imp1$psi_depressao_30d_t0=='sim') +
 # (covariates_imp1$psi_dificuldade_dormir_30d_t0=='sim')
 #
 # covariates_imp1$psi_ansiedade_30d_t0 = NULL
 # covariates_imp1$psi_depressao_30d_t0 = NULL
 # covariates_imp1$psi_dificuldade_dormir_30d_t0 = NULL

 dat_fit = cbind(df[,c('duracao_tratamento_weeks', 'event')],
 covariates_imp1)

 # dat_fit$duracao_tratamento_weeks2 = dat_fit$duracao_tratamento_weeks +
 # runif(nrow(dat_fit), 0, 1/7)

 #dat_fit$substancia_cocacrack = dat_fit$substancia == 'cocainacrack'
 dat_fit$substancia_outras = dat_fit$substancia == 'outras'
 #dat_fit$substancia_canabinoides = dat_fit$substancia == 'canabinoides'
 dat_fit$ano_t0 = (dat_fit$ano_t0 - 2000)/10

 fit <- coxph(
 Surv(duracao_tratamento_weeks, event) ~
 escolaridade+raca+ #psi_comorbidade_score+
 psi_tentativa_suicidio_vida_t0 +#substancia_cocacrack +
 #tt(substancia_cocacrack) +
 substancia + #tt(substancia_outras) +
 ano_t0+ #tt(ano_t0)+
 abuso_sex_conhecido_t0+faixa_renda_t0 + faixa_idade,
 #tt = function(x, t, ...) {x * t},
 data = dat_fit)

 dat_fits[[i]] = dat_fit
 results[[i]] <- fit
 }

 ### residuos

 ph_test = list()
 for (i in 1:num_imp) {
 ph_test[[i]] <- cox.zph(results[[i]], terms=F)$table
 }

 test_res = do.call(rbind, ph_test)

 schoenfeld_test_results_merged = list()
 i = 1
 for (var in unique(rownames(test_res))) {
 tmp = test_res[rownames(test_res) == var,]


 schoenfeld_test_results_merged[[i]] =
 miceadds::micombine.chisquare(dk = tmp[,'chisq'],
 df = unique(tmp[,'df']), display = F)
 i = i+1
 }

 schoenfeld_test_results_merged = do.call(rbind, schoenfeld_test_results_merged)
 rownames(schoenfeld_test_results_merged) = rownames(ph_test[[1]])

 # Extract variable names excluding 'GLOBAL'
 variables <- rownames(schoenfeld_test_results_merged)
 variables <- variables[variables != "GLOBAL"]

 ph_test = list()
 for (i in 1:num_imp) {
 ph_test[[i]] <- cox.zph(results[[i]], terms=F)
 }

 # Prepare averaged residuals for each covariate
 avg_residuals <- list()

 var = variables[1]
 for (var in variables) {
 # Extract residuals for current variable from all imputations
 y_list <- lapply(ph_test, function(test) test$y[, var])
 # Combine into matrix and compute row means
 y_matrix <- do.call(cbind, y_list)
 y_mean <- rowMeans(y_matrix)
 # Get time values (assumed consistent across imputations)
 x <- ph_test[[1]]$time
 # Store results
 avg_residuals[[var]] <- data.frame(x = x, y = y_mean, variable = var)
 }

 # Combine into single dataframe
 avg_residuals_df <- do.call(rbind, avg_residuals)

 # Prepare p-value annotations
 annotations <- data.frame(
 variable = variables,
 label = sprintf("p = %.4f", schoenfeld_test_results_merged[variables, "p"])
 )

 # Generate plot
 library(ggplot2)

 newvarmap = tribble(
 ~variable, ~new,
 'escolaridadecomplete primary', 'Education Level: Complete Primary',
 'escolaridadecomplete secondary', 'Education Level: Complete Secondary',
 'escolaridadecomplete tertiary', 'Education Level: Complete Tertiary',
 'substanciacocainacrack', 'Main substance: Cocaine/Crack',
 'racaindigenaamarela', 'Race: Indigenous and Asian',
 'racapretaparda', 'Race: Black and Brown',
 # 'psi_comorbidade_score', 'Pyschiatric Commorbidity Score',
 'psi_tentativa_suicidio_vida_t0sim', 'Previous suicide attempt',
 'substanciacanabinoides', 'Main substance: Cannabinoids',
 'substanciaoutras', 'Main substance: Others',
 'ano_t0', 'Year of treatment start',
 'abuso_sex_conhecido_t0sim', 'Sexual abuse',
 'faixa_renda_t0TRUE', 'Income',
 'faixa_idade26-59', 'Age Stratum: 26-59',
 'faixa_idade60+', 'Age Stratum: 60+',
 )

 avg_residuals_df_plt = left_join(avg_residuals_df, newvarmap)
 annotations_plt = left_join(annotations, newvarmap)

 ggplot(avg_residuals_df_plt, aes(x = x, y = y)) +
 geom_point(alpha = 0.6) +
 geom_smooth(method = "loess", color = "red", se = TRUE) +
 facet_wrap(~ new, scales = "free_y", ncol=3) +
 geom_text(data = annotations_plt,
 aes(label = label),
 x = Inf, y = Inf,
 hjust = .9, vjust = -0.5,#.5,
 size = 3.5, color = "blue") +
 labs(x = "Time",
 y = "Averaged Schoenfeld Residuals",
 title = paste0(
 "Schoenfeld Residuals with Proportional Hazards Test (Global Test: p = ",
 round(schoenfeld_test_results_merged['GLOBAL','p'],5),
 ')')
 ) +
 theme_minimal() + coord_cartesian(clip='off')

*Figure 2*

#####
 ##### kaplan meier
 #####

 library(tidyverse)
 library(survival)
 library(survminer)

 df <- read.csv('dados_final/dados_consolidados_t0.csv')

 # preparar covariaveis
 df$event <- df$status == 'abandono'

 # n inicial = 854
 nrow(df)

 # com indicador de evento = 790 (o resto nÃ£o tem status)
 df = df[!is.na(df$event),]
 nrow(df)

 # 667 que passaram da triagem
 df = df[-which(df$status == 'abandono na triagem'),]
 nrow(df)

 df$duracao_tratamento_weeks = df$duracao_tratamento/7

 # tirando quem abandonou, mas nÃ£o tem data de abandono = 651
 df = df[!(is.na(df$duracao_tratamento_weeks) & df$event),]
 nrow(df)

 # colocando duracao tratamento de quem ta em tratamento
 # = 10-06-24 - entrada
 df$duracao_tratamento_weeks[df$status == 'em tratamento'] =
 (as.numeric(as.Date('2024-06-10') -
 as.Date(df$sociodem_data_t0[df$status == 'em tratamento'])))/7

 # varias altas sem data, n = 641
 df[df$status=='alta mÃ©dica'&is.na(df$duracao_tratamento_weeks),]$id_promud
 df = df[!is.na(df$duracao_tratamento_weeks),]
 nrow(df)

 # janela de dois anos
 df$event[which(df$duracao_tratamento_weeks>104)] = F
 df$duracao_tratamento_weeks[df$duracao_tratamento_weeks>104] = 104

 # TODO: ver qual o problema com esses tratamentos
 # que tem duracao negativa
 # 3 com duracao de tratamento negativa, n= 638
 df = df[-which(df$duracao_tratamento_weeks <0),]
 nrow(df)

 # faixa etaria
 df$stratum = ifelse(
 df$sociodem_idade_t0 < 26, "18-25",
 ifelse(
 df$sociodem_idade_t0 <= 59, "26-59",
 ifelse(
 df$sociodem_idade_t0 >= 60, "60+",
 NA
 )
 )
 )

 fit = survfit(Surv(duracao_tratamento_weeks, event) ~ stratum, data = df,
 conf.type = 'log-log')

 ggsurvplot(fit, data = df, pval = TRUE,
 ylab = "Treatment retention probability",
 xlab = "Treatment duration (weeks)",
 legend.title = "", # Remove the legend title
 legend.labs = levels(df$stratum), conf.int = T)

 summary_fit <- summary(fit, times = c(25, 52, 104), extend = TRUE)

 # Extract results for each group:
 surv_table <- data.frame(
 time = summary_fit$time,
 strata = summary_fit$strata,
 survival = round(summary_fit$surv,2),
 lower_ci = round(summary_fit$lower,2),
 upper_ci = round(summary_fit$upper,2)
 )

*Figure 3*

################# MODELO VARIANDO NO TEMPO

 results <- list()
 dat_fits <- list()

 for (i in 1:num_imp) {
 covariates_imp1 = complete(covariates_imp, i)

 # covariates_imp1$psi_comorbidade_score =
 # (covariates_imp1$psi_ansiedade_30d_t0=='sim') +
 # (covariates_imp1$psi_depressao_30d_t0=='sim') +
 # (covariates_imp1$psi_dificuldade_dormir_30d_t0=='sim')
 #
 # covariates_imp1$psi_ansiedade_30d_t0 = NULL
 # covariates_imp1$psi_depressao_30d_t0 = NULL
 # covariates_imp1$psi_dificuldade_dormir_30d_t0 = NULL

 dat_fit = cbind(df[,c('duracao_tratamento_weeks', 'event')],
 covariates_imp1)

 # dat_fit$duracao_tratamento_weeks2 = dat_fit$duracao_tratamento_weeks +
 # runif(nrow(dat_fit), 0, 1/7)

 dat_fit$substancia_cocacrack = dat_fit$substancia == 'cocainacrack'
 dat_fit$substancia_outras = dat_fit$substancia == 'outras'
 dat_fit$substancia_canabinoides = dat_fit$substancia == 'canabinoides'
 dat_fit$ano_t0 = (dat_fit$ano_t0 - 2000)/10

 fit <- coxph(
 Surv(duracao_tratamento_weeks, event) ~
 escolaridade+raca+#psi_comorbidade_score+
 psi_tentativa_suicidio_vida_t0 + substancia_cocacrack +
 tt(substancia_cocacrack) +
 substancia_outras + tt(substancia_outras) +
 ano_t0+ tt(ano_t0)+ substancia_canabinoides +
 abuso_sex_conhecido_t0+faixa_renda_t0 + faixa_idade,
 tt = function(x, t, ...) {x * t},
 data = dat_fit)

 dat_fits[[i]] = dat_fit
 results[[i]] <- fit
 }

 result_table = pool(results)

 pooled = result_table$pooled

 data.frame(
 covariavel = pooled$term,
 coef = round(pooled$estimate,2),
 se = round(sqrt(pooled$t),2),
 HR = round(exp(pooled$estimate),2),
 stat = round(pooled$estimate/sqrt(pooled$t),2),
 df = round(pooled$df, 1),
 pval = round(2*pt(-abs(pooled$estimate/sqrt(pooled$t)), df=pooled$df),3)
 )

 # covariavel coef se HR stat df pval
 # 1 escolaridadecomplete primary -0.02 0.19 0.98 -0.10 195.2 0.919
 # 2 escolaridadecomplete secondary -0.21 0.16 0.81 -1.28 215.6 0.200
 # 3 escolaridadecomplete tertiary -0.41 0.20 0.66 -2.06 254.3 0.041
 # 4 racaoutra -0.23 0.13 0.80 -1.73 244.9 0.085
 # 5 psi_comorbidade_score -0.05 0.08 0.95 -0.61 59.8 0.542
 # 6 psi_tentativa_suicidio_vida_t0sim -0.07 0.13 0.93 -0.57 128.1 0.569
 # 7 substancia_cocacrackTRUE 0.43 0.19 1.53 2.28 353.9 0.023
 # 8 tt(substancia_cocacrack) -0.01 0.00 ---- -1.90 347.7 0.058
 # 9 substancia_outrasTRUE -0.80 0.32 0.45 -2.50 345.3 0.013
 # 10 tt(substancia_outras) 0.02 0.01 ---- 2.91 352.3 0.004
 # 11 ano_t0 0.43 0.11 1.53 3.84 337.6 0.000
 # 12 tt(ano_t0) -0.01 0.00 ---- -3.31 372.6 0.001
 # 13 abuso_sex_conhecido_t0sim -0.12 0.16 0.89 -0.74 71.0 0.460
 # 14 faixa_renda_t0TRUE -0.06 0.14 0.94 -0.47 93.0 0.638
 # 15 faixa_idade26-59 -0.41 0.15 0.66 -2.74 355.8 0.006
 # 16 faixa_idade60+ -1.21 0.30 0.30 -3.99 364.7 0.000


 ########## pegar estimativas para plot...
 cov_matrix = Reduce(`+`, lapply(results, function(x) x$var))/num_imp
 sds <- sqrt(diag(cov_matrix))
 D <- diag(1/sds)
 cor_matrix <- D %*% cov_matrix %*% D
 D_new <- diag(sqrt(pooled$t))
 cov_matrix <- D_new %*% cor_matrix %*% D_new

 ########## PLOT HR: substancia

 # data for Crack/Cocaine

 idx <- which(pooled$term %in% c("substancia_cocacrackTRUE", "tt(substancia_cocacrack)"))
 beta <- pooled$estimate[idx[1]]
 gamma <- pooled$estimate[idx[2]]
 cov_mat <- cov_matrix[idx, idx]

 time_points <- seq(0, max(dat_fit$duracao_tratamento_weeks), length.out = 100)
 log_hr <- beta + gamma * time_points
 var_log_hr <- cov_mat[1,1] + (time_points^2 * cov_mat[2,2]) + (2 * time_points * cov_mat[1,2])
 se_log_hr <- sqrt(var_log_hr)

 HR <- exp(log_hr)
 lower_hr <- exp(log_hr - 1.96 * se_log_hr)
 upper_hr <- exp(log_hr + 1.96 * se_log_hr)

 df_crack <- data.frame(t = time_points, m = HR, l = lower_hr, u = upper_hr,
 substance = "Crack/Cocaine")

 # data for Other Substances

 idx <- which(pooled$term %in% c("substancia_outrasTRUE", "tt(substancia_outras)"))
 beta <- pooled$estimate[idx[1]]
 gamma <- pooled$estimate[idx[2]]
 cov_mat <- cov_matrix[idx, idx]

 log_hr <- beta + gamma * time_points
 var_log_hr <- cov_mat[1,1] + (time_points^2 * cov_mat[2,2]) + (2 * time_points * cov_mat[1,2])
 se_log_hr <- sqrt(var_log_hr)

 HR <- exp(log_hr)
 lower_hr <- exp(log_hr - 1.96 * se_log_hr)
 upper_hr <- exp(log_hr + 1.96 * se_log_hr)
 upper_hr = ifelse(upper_hr > 4, 4, upper_hr)

 df_outras <- data.frame(t = time_points, m = HR, l = lower_hr, u = upper_hr,
 substance = "Other substances")

 # Combine data
 combined_df <- rbind(df_crack, df_outras)

 # Create combined plot
 p1 = ggplot(combined_df, aes(x = t)) +
 geom_line(aes(y = 1, linetype = "Alcohol reference")) + # Add reference line with legend entry
 geom_line(aes(y = m, color = substance)) +
 geom_ribbon(aes(ymin = l, ymax = u, fill = substance), alpha = 0.2) +
 theme_bw() + ylim(0,4) +
 labs(x = "Treatment duration (weeks)", y = "Hazard Ratio",
 title = "Substance of Choice vs Alcohol") +
 scale_color_manual(values = c("Crack/Cocaine" = "#E69F00",
 "Other substances" = "#56B4E9")) +
 scale_fill_manual(values = c("Crack/Cocaine" = "#E69F00",
 "Other substances" = "#56B4E9")) +
 scale_linetype_manual(values = "dashed") +
 guides(
 color = guide_legend(
 title = "Comparison",
 override.aes = list(linetype = c("solid", "solid")) # Force solid lines in legend
 ),
 fill = "none", # Hide fill legend (redundant with color)
 linetype = guide_legend(
 title = NULL,
 override.aes = list(color = "black") # Show reference line in black
 )
 ) +
 theme(legend.spacing.y = unit(0, "mm")) # Tighten legend spacing
 p1
 ########## PLOT HR: Ano
 idx = which(pooled$term %in% c("ano_t0", "tt(ano_t0)"))
 beta <- pooled$estimate[idx[1]]
 gamma <- pooled$estimate[idx[2]]
 cov_mat = cov_matrix[idx, idx] #covariance matrix 2x2 for beta and gamma

 # Define time points
 time_points <- seq(0, max(dat_fit$duracao_tratamento_weeks), length.out = 100)

 # Calculate hazard ratio (HR) over time
 log_hr = beta + gamma * time_points
 var_log_hr <- cov_mat[1,1] + (time_points^2 * cov_mat[2,2]) +
 (2 * time_points * cov_mat[1,2])
 se_log_hr <- sqrt(var_log_hr)

 # Calculate HR and 95% CIs
 HR <- exp(log_hr)
 lower_hr <- exp(log_hr - 1.96 * se_log_hr)
 upper_hr <- exp(log_hr + 1.96 * se_log_hr)

 p2 = data.frame(t = time_points, m = HR, l = lower_hr, u = upper_hr) %>%
 ggplot(aes(x=t)) + geom_line(aes(y=1), linetype=3) +
 geom_line(aes(y=m)) +
 geom_ribbon(aes(ymin=l,ymax=u), alpha=.1) +
 theme_bw() + xlab('Treatment duration (weeks)') + ylab('Hazard Ratio') +
 ggtitle('10-year increase in treatment year')
 ##########

 p2 + p1
