# Supplementary material for: Gone Too Soon: Higher Pretreatment and Treatment Dropout Among Emerging Adults in a Women‐Specific Outpatient Treatment Service for Substance Use Disorders in Brazil
Source: Early Interv Psychiatry. 2026 Jan 11;20(1):e70133. doi: 10.1111/eip.70133 (PMC12791191; doi:10.1111/eip.70133)
Supplement: Supplementary file 2 — Figure S1: Schoenfeld residual plots assessing the proportional hazards assumption for covariates in the Cox model (treatment group). [file EIP-20-0-s002.docx]

**Supplementary Material**

**Supplementary Figure 1.** Schoenfeld residual plots assessing the proportional hazards assumption for covariates in the Cox model (treatment group).


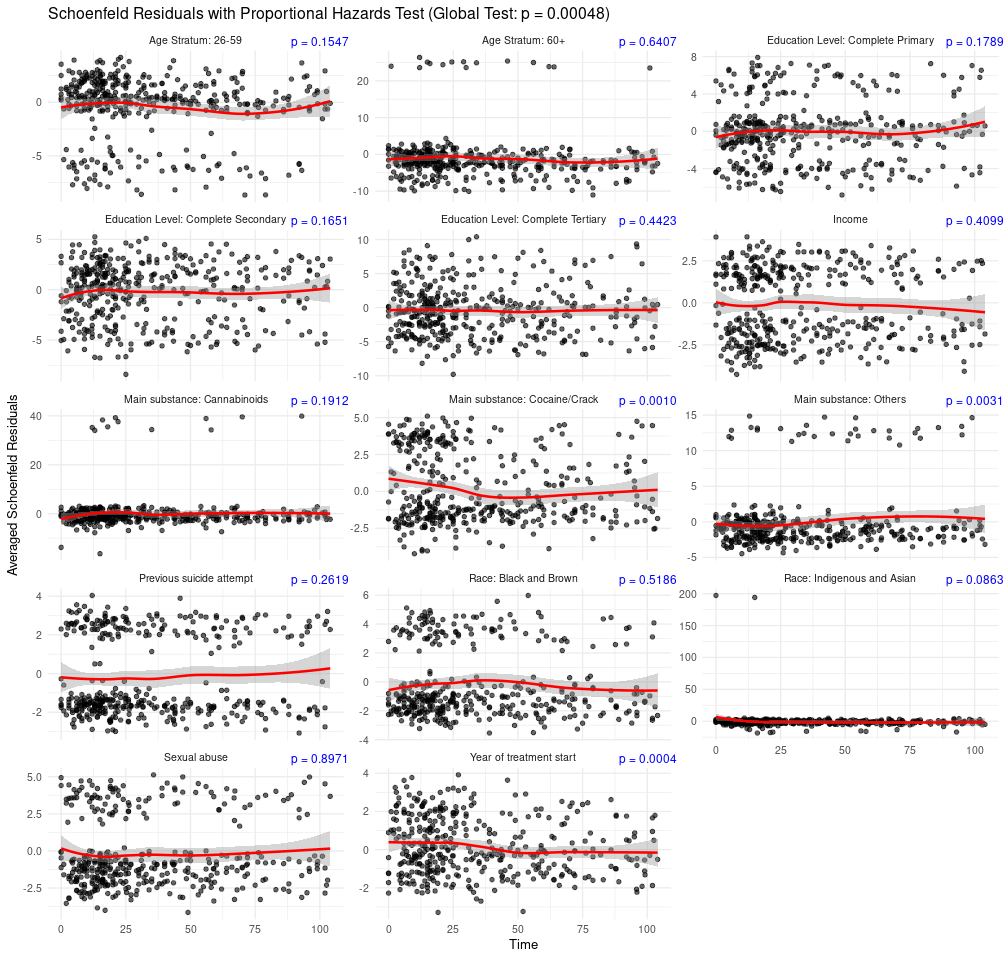


Scaled Schoenfeld residuals are plotted against time for each covariate included in the Cox proportional hazards model. The red lines represent smoothed curves fitted to the residuals, with deviations from horizontality indicating potential violations of the proportional hazards assumption. P-values from individual tests are shown in blue, and the global test of proportionality was statistically significant (p = 0.00048). Notably, non-proportional hazards were observed for year of treatment start (“year at admission”) and main substance of use: cocaine/crack, and others, supporting the inclusion of time-varying effects for these covariates in the final model.
